# Comprehensive Study on the Acrylamide Content of High Thermally Processed Foods

**DOI:** 10.1155/2021/6258508

**Published:** 2021-02-23

**Authors:** Dilini N. Perera, Geeth G. Hewavitharana, S. B. Navaratne

**Affiliations:** Department of Food Science and Technology, University of Sri Jayewardenepura, Nugegoda 10250, Sri Lanka

## Abstract

Acrylamide (AA) formation in starch-based processed foods at elevated temperatures is a serious health issue as it is a toxic and carcinogenic substance. However, the formation of more AA entangles with modern-day fast food industries, and a considerable amount of this ingredient is being consumed by fast food eaters inadvertently throughout the world. This article reviews the factors responsible for AA formation pathways, investigation techniques of AA, toxicity, and health-related issues followed by mitigation methods that have been studied in the past few decades comprehensively. Predominantly, AA and hydroxymethylfurfural (HMF) are produced via the Maillard reaction and can be highlighted as the major heat-induced toxins formulated in bread and bakery products. Epidemiological studies have shown that there is a strong relationship between AA accumulation in the body and the increased risk of cancers. The scientific community is still in a dearth of technology in producing AA-free starch-protein-fat-based thermally processed food products. Therefore, this paper may facilitate the food scientists to their endeavor in developing mitigation techniques pertaining to the formation of AA and HMF in baked foods in the future.

## 1. Introduction

Food processing generally includes the basic preparation that is used to convert raw ingredients into food or food into other forms of consumption. Numerous processing stages such as washing, cutting, drying, frying, fermenting, cooking, and many more are involved as a single operation or a combination. During these processing steps, some toxic compounds can be formed [1]. AA is one among such heat-induced toxic substance which may be developed in starchy foods at high-temperature processing primly via the Milliard reaction pathway [2]. It can be formed in baked, fried, grilled, toasted, and roasted foods [3, 4].

AA (C_3_H_5_NO; prop-2-enamide) is a colorless, odorless, and water-soluble compound and thermally decomposes to CO_2_, CO, NO_2_, and NH_3_ [5]. In 2002, the Swedish National Food Authority and Stockholm University have discovered that AA in foods was processed above the temperature of 120°C [6]. Based on its toxicity, different tolerable daily intakes were estimated. To avoid AA neurotoxicity, intake is estimated at 40 *μ*g/kg bw/day, and to avoid its carcinogenic effects, 2.6 *μ*g/kg bw/day [7, 8]. Different factors can be investigated with respect to the formation of AA in foods, and consequently, formation mechanisms, several mitigation approaches, and future perspectives were discussed in this review.

## 2. Factors Affecting Acrylamide Formation

Food composition and processing conditions are the two main factors that directly affect AA formation. Food composition includes the availability of amino acids, presence of sugars, pH, and moisture of the food [9]. Reducing sugar and amino acids are the starting reactants. Asparagine is the key amino acid, and the presence of mono or disaccharides such as glucose, fructose, and sucrose directly affects the formation of AA [10]. The reactivity of sugars differs according to their chain length; sugars that have shorter carbon chains lead to high AA formation. Thus, pentoses are more reactive than hexoses and disaccharides in yielding brown color in the Maillard reaction [11, 12]. Also, aldo sugars are more reactive than keto sugars, and among isomeric sugars, ribose is more reactive than xylose in AA formation [13, 14].

When comparing the availability of reducing sugars and amino acids for AA formation in potatoes, cereal products, and coffee, some limiting factors for the reaction can be found. Asparagine and glutamine are abundant in potatoes and lack in reducing sugars [15]. For cereal products, asparagine is limited [16], and coffee is rich in both asparagine and sucrose, which during roasting sucrose fragmented into glucose and fructose [17]. Then, AA was formed in those food products. Water content in the food matrix can also affect AA formation. Water activity would be below 0.8 which facilitates the formation of AA, whereas the AA formation is high at the water activity 0.4 or below [8]. However, AA can be removed from heated foods such as biscuits and potato chips with an increase in water activity [18]. Anese et al. [19] reported that 32% of acrylamide can be removed in cookies at water activity 0.83, whereas it is 12% at water activity 0.12. In potato chips, acrylamide removal zeroes at the water activity 0.26 while it is 20% at water activity 0.95.

The pH of the medium is another factor that is strongly influenced by the Maillard reaction and then leads to AA formation. Acidic conditions disrupt the AA formation, thus lowering the pH using organic acids in the food system which resulted in reduced acrylamide generation by protonating the *α*-amino group of asparagine, which subsequently cannot engage in nucleophilic addition reactions with carbonyl sources. The correlation between pH decrease and acrylamide reduction varies among products due to multiple factors of different starting pH values of the products [20].

Since AA formation is initiated by heat, the effect of processing temperature and time in relation to AA formation is critically important. According to the Arrhenius equation, temperature dependence of chemical reactions is often expressed as the activation energy (Ea) [21]. The higher the Ea, the more temperature-dependent the reaction rate. The Ea for the Maillard reaction which is directly proportional to AA formation has been reported within a wide range, 10–160 kJ mol^−1^ [22], depending on other factors that affect the reaction has been measured. Matthäus et al. [23] reported that time duration is a key factor especially frying temperature at or above 175°C. They reported a rapid increase in AA concentration at 180–190°C compared to lower temperatures (150°C and 175°C).

## 3. Formation of Acrylamide

AA is not a component of food; it is formed during heat processing. The reaction routes for the formation of AA during processing can be explained based on different hypotheses that were found to be most relevant and probable in a processing situation. The main precursors reported for AA formation included 3-aminopropionamide (3APA), decarboxylated Schiff base, decarboxylated Amadori product, acrylic acid, and acrolein [4, 20, 23]. According to the literature, there are two most accepted pathways; the Maillard reaction pathway and the acrolein pathway [8] which were explained in the following section.

**Figure 1:**
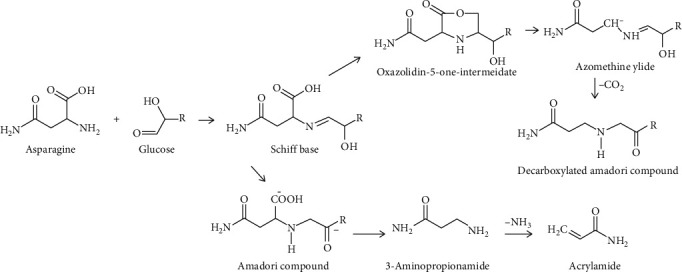
The major pathway for AA formation in food [30].

The formation of AA via the Maillard reaction is known as the major pathway; the reaction between free amino acids such as asparagine and reducing sugars generates a Schiff base by the removal of water from sugar–protein conjugate, and further decarboxylates through the formation of oxazolidine-5-one. The decarboxylated product decays to AA through the removal of imine or after hydrolyzation to form 3-APA [24] (Figure 1). Zhang et al. [25] reported that this asparagine pathway was mainly accountable for AA formation in cooked foods' subsequent condensation with reducing sugars. In model studies, it has been shown that *α*-hydroxy carbonyls are more efficient than *α*-dicarbonyls in forming AA [26]. Furthermore, Eriksson [27] reported that fructose increases the AA content by about two times in comparison with other reducing sugars because it contains two *α*-hydroxylic groups rather than one as is the case with other sugars such as glucose.

Besides, 3-APA can be formed AA in the absence of reducing sugars. Based on structural considerations, asparagine alone can be changed into AA through decarboxylation and deamination reactions under thermal processing [27, 28]. Nevertheless, the key product of thermal degradation of asparagine is maleimide; however, it prevents the formation of AA due to rapid intramolecular cyclization reactions [29].

**Figure 2:**
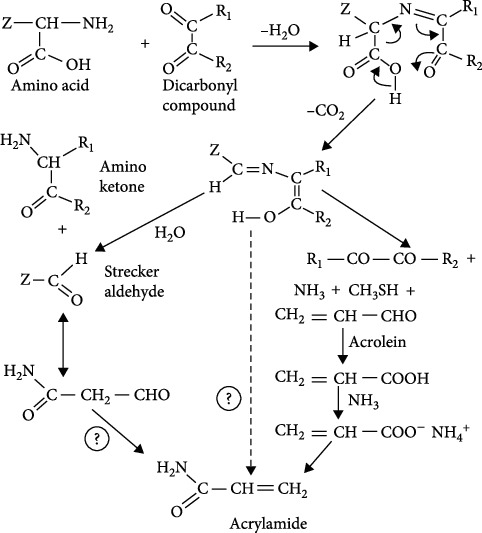
Pathways for the formation of AA after Strecker degradation of amino acids asparagine and methionine. In asparagine, the side chain Z is –CH_2_CONH_2_; in methionine, it is –CH_2_CH_2_SCH_3_ [26].

Apart from the major pathway, the Strecker reaction of asparagine generates direct intermediate as Strecker aldehydes [31] (Figure 2). Mottram et al. [32] reported that AA was formed during heat processing associated with amino acids and reducing sugars; hence, most of the flavors and color generated from intermediates have formed the Strecker degradation of amino acids during baking and roasting.

**Figure 3:**
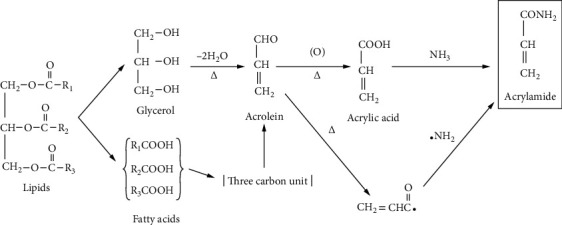
Acrolein pathway for AA formation in foods [8].

The acrolein pathway is the second most accepted way of AA formation. AA is generated from oil when heated at temperatures above the smoke point. Initially, oil is hydrolyzed into glycerol and fatty acids followed by the elimination of water from glycerol and produced acrolein via heterolytic acid-catalyzed carbonium ion mechanism [33, 34] (Figure 3). Oils, which are highly unsaturated and have a lower smoke point, lead to the formation of acrolein [35].

**Figure 4:**
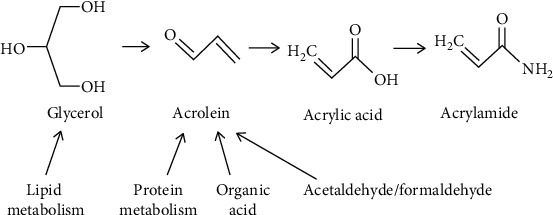
Possible ways of acrolein formation [30].

There are some other possible ways of acrolein formation such as degradation of amino acids and proteins, degradation of carbohydrates, and decarboxylation of an organic acid such as lactic acid, citric acid, and malic acid [33] (Figure 4), hence resulting in acrolein which further reacts with NH_3_ to produce AA, but it is limited to some extent because carbonyl compounds such as reducing sugars are more active with asparagine than them [4].

### 3.1. Determination of Acrylamide

**Figure 5:**
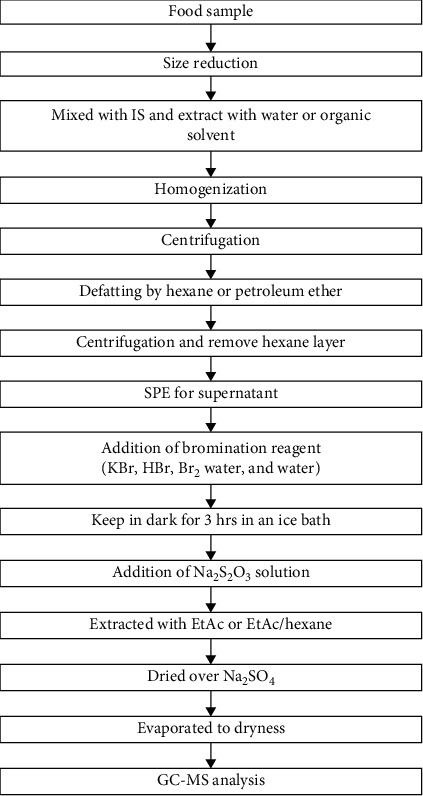
Sample preparation flow chart.

Analytical methods are mainly based on the mass spectrometry (MS) technique coupled with either liquid chromatography (LC) or gas chromatography (GC) [36]. Wenzl et al. [37] have reported that the performance of the LC method was greater than the GC method. Since AA is a tiny, extremely polar molecule, its extraction and analysis are difficult. Sample preparation had a great influence on the accurate quantification of AA. Since AA may be firmly enclosed and concentrated at the surface, the whole food sample should be homogenized thoroughly before a sample is taken for analysis. Thereafter, internal standard (IS), defatting, extraction, concentration, and cleanup (in some methods also derivatization) were added, and chromatography/detection were followed [37–39]. AA quantification can be done based on the external and internal standard method. An IS should be added to the extraction, to overcome the poor reproducibility and recovery yield of AA. Thus, it improves the accuracy and precision and reduces the quantitative issues associated with ion suppression [38].  Figure 5 illustrates the basic flow chart of sample preparation for GC-MS analysis.

There is no fully accepted extraction method of AA from various food. AA is readily soluble in water, lower alcohols, and other polar organic solvents [40]. Extraction can be done with water or with an organic solvent. The water reduces the solubility of hydrophobic compounds and also minimizes the extraction of other unwanted compounds such as salts, proteins, and carbohydrates, which might interfere with the detection and degenerate the chromatographic system in the food products. Starch, protein, and fat should be removed after extraction with water [41, 42]. Consequently, defatting must be done using organic solvents such as hexane, dichloromethane, petroleum ether, and cyclohexane, because high-fat content leads to overlapping peaks of AA or disruptive the analytical column of GC [36]. For high-protein samples, deproteinization can be done with acetonitrile (ACN), methanol, ethanol, acetone, sodium chloride, or Carrez reagents, and filtration through a cutoff filter [12, 40]. Furthermore, to achieve a better penetration of the solvents, elevated temperatures are used for swelling of the food matrix [43]. Apart from water, organic solvents such as n-propanol, methanol, acetone, and ACN can also be used for AA extraction [36]. Organic solvents give clear solution mostly without centrifugation, and it extracts lipids and can be evaporated easily.

Moreover, a quick and automated solvent extraction method with pure water and formic acid was introduced by Cavalli et al. [44]. They reported that pure water extracts exhibited lower recoveries than the formic acid, but the formic acid extracts had lowered the stability. Extraction can be completed by single-stage or a combination of stages. Single-stage extraction has been substantially applied to extract AA from compact food matrices using slight variations in extraction conditions [43]; extraction solvent type, solvent-to-sample ratio, extraction time, temperature, particle size, defatting, homogenization, and the application of mechanical forces affect AA extraction yield [42, 44]. AA also can extract through the salt-out technique from the aqueous mixture and concentrated through rotary evaporation. In this method, ethyl acetate (EtAc) was used as it prevents the extraction of other interfering substances which include salt, sugars, starches, and amino acids [45].

Apart from conventional methods, novel sample preparation methods are also used for the analysis. The dispersive solid-phase extraction (DSPE) method leads to rapid, easy, economical, and safe extractions [45]. Matrix solid-phase dispersion (MSPD) offers a porous structure that the solvent can penetrate easily [46]. Pressurized fluid extraction (PFE) comprises extraction with liquid solvents at higher temperatures and pressures which have already been used for the extraction of AA from foods [47]. Other than that, solvent-free sample preparation techniques such as solid-phase microextraction (SPME) can be used in the analysis of AA [48]. In this method, the extrication of analytes from the sample followed by fiber coating and then desorption of the extract from the fiber into the analytical instrument where the sample components are thermally desorbed generally in GC were performed [36, 49]. Furthermore, the AA could be extracted either by directly submerging the solid phase in an aqueous solution (DISPME) or submerging the vapor phase above the aqueous solution, known as headspace (HS-SPME) [50].

Derivatization is done to reduce the polarity of AA, expand the retention time, regulate peaks, and ensure the optimization of corresponding parameters [51]. The GC-MS analysis based on bromination has advantages such as bromination produces fewer polar molecules than the natural AA, which is easily extracted by EtAc or hexane, and eliminating many water-soluble components enlarges AA molecular weight, which leads to improved mass spectrometry (MS) characteristics, giving several ions to monitor and confirm, and relatively more volatile derivatization product improves GC characteristics [52]. Commonly, a bromine water solution with HBr and KBr or a mixture of KBr and KBrO_3_ is added into the pretreated AA extracts. Tables 1 and 2 indicate a summary of reported AA extraction and derivatization methods and AA content in different food varieties respectively.

Bromination is carried out at a refrigerator or on ice in a dark place [36]. Methacrylamide or N,N-dimethylacrylamide could be used as IS produce a derivative of 2,3-dibromo-2-methylpropionamide. Thus, the end derivative 2,3-dibromopropionamide is rather unstable and might degrade in the GC injector to 2bromopropenamide [53]. After the derivatization, the excess bromine is removed by titration with sodium thiosulfate (Na_2_S_2_O_3_). The brominated AA is less polar compared with the extracted compound, which is more soluble in nonpolar organic solvents [54]. Frequently, EtAc or a combination of EtAc and cyclohexane is used for the extraction of the analyte from the aqueous phase [39]. Phase separation is frequently conducted by centrifugation of the sample. Moreover, cleanup was carried out by passing organic extract through a silica-gel cartridge [26]. Due to the high-water absorptivity of silica, EtAc must be dried or exchanged with cyclohexane to avoid variations of the silica activity. Current studies reported that the remaining water and water-soluble coextractant will interfere with the results and can be eliminated by drying the extract Na_2_SO_4_ [36]. Generally, midpolar to polar columns are used for bromo derivatives in GC analysis [4]. Apart from the bromination of AA, other techniques such as HS-SPME can be done by silylation with N,O-bis(trimethylsilyl) trifluoroacetamide (BSTFA) to form the volatile N,O-bis(trimethylsilyl) acrylamide (BTMSA) [36]. Then, the generated BTMSA is extracted over the silylation reaction with the use of a poly(dimethylsiloxane) SPME fiber from the headspace [54].

## 4. Toxicity of Acrylamides

Scientists have focused on the different health effects of AA due to its high toxicity. It could be inducing neurotoxicity, genotoxicity, carcinogenicity, hepatotoxicity, and reproductive toxicity to humans and animals [74, 75]. AA and its toxicity have been evaluated by the International Agency for Research on Cancer (IARC) and by the Joint FAO/WHO Expert Committee on Food Additive (WHO/JECFA) [76, 77]. According to their findings, average human intake is valued to be 0.4 *μ*g/kg bw/day from two years of age, though consumption may vary generally from 0.3 *μ*g/kg bw/day to 5 *μ*g/kg bw/day. The estimate of average daily human intake was 1 *μ*g/kg bw/day, and it can be 4 *μ*g/kg bw/day for high consumers [77]. Several toxic effects of AA are summarized in the following section.

AA is a neurotoxicant, which can be accidental intoxications or chronic occupational exposure, and affected central nervous system (CNS) and peripheral nervous system (PNS) in rodents and humans [78]. The toxic effect depends on exposure time and dosage. Highly exposed workers in china were shown to that peripheral neuropathy symptom such as skeletal muscle weakness, tingling of hands and feet, and ataxia. Thus, cerebellar Purkinje cells and distal axons in the central nervous system were also affected [79]. Furthermore, the degradation of nerve terminals which act as a primary site of AA action leads to the weakening of cognitive functions and damage to the cerebral cortex, thalamus, and hippocampus [80]. According to the studies in rats and mice, NOEL (no observable effect level) for neurotoxicity was reported as 0.2 to 10 mg/kg bw/day [81].

AA is a directly acting clastogen, which causes abnormality in genes that leads to genotoxicity. In mammalian cells, metabolic conversion of AA into glycidamide (GA) causes mutagenic effects at the HPRT locus, and also, AA can act as a Michael acceptor and form adducts with thiol, hydroxyl, or amino groups and nucleophilic centers in DNA. AA also induces germ cell mutations [29, 82]. It was recently found that GA significantly induces micronuclei, impairs cell propagation kinetics, and decreases cell viability at high concentrations by mechanisms not involving oxidative stress. Further, it is confirming that human mammary cells are susceptible to GA toxicity [83, 84].

In 1994, the IARC had been classified AA as possible carcinogenic to humans based on the positive bioassay results in rodents and other evidence by the genotoxic effect of GA [77, 85]. Formation of covalent adducts with DNA and hemoglobin, induction of chromosomal aberrations in somatic cells of rodents in vivo, chromatic mutations, and chromosomal abnormalities in cultured and germ cells in vitro, and induction of cell transformation in mouse cell lines indicated the carcinogenic effect [30]. Important laboratory experiments of rodents illustrated that AA is carcinogenic, causing tumors in the lungs, skin, brain, mammary gland, thyroid gland, and uterus [81, 85]. Occupational exposure to AA seems to be the most affected to human cancer risk.

The reproductive and developmental toxicity of AA has been demonstrated with laboratory animals. No observed adverse effect level was reported as 2 to 5 mg/kg/day in rats [86]. After injection of exogenous AA to rodents, disturbing mating, reduced fertility rates, increased resorptions of fetuses, reduced litter size in pregnant females, abnormal sperm, and low sperm count in males were shown [87]. These effects are associated with gene mutilation, alkylation of sulfhydryl (SH) groups, reduction of glutathione (GSH), and DNA damages [88]. However, there was less evidence for the potential effects of reproductive and developmental toxicity for humans.

Some researchers have discussed the hepatotoxicity effect of AA due to oxidative stress. Based on the experimental results, exposure to a high dose of 25 mg/kg for 21 days caused a significant decrease in liver GSH level and total antioxidant in adult rats [89]. Furthermore, the accumulation of AA also resulted in increased serum level of liver enzymes, a decrease in superoxide dismutase and catalase activities, and increased total oxidants and malondialdehyde levels [8]. However, hepatotoxicity in humans is still very less.

## 5. Mitigation Techniques of Acrylamide in Food

Various AA mitigation techniques have been described in the literature, and a summary of those approaches is discussed in the following section. According to findings, this can be done by maintaining low AA formation throughout the heat processing and removal of already formed AA in finished products. Low AA formation could be expected through appropriate processing conditions, using agronomic and genetic approaches, pretreatments, and by adding specific additives, plant extracts, competitive inhibitors, and enzymes [1, 11, 24, 30, 75, 84].

### 5.1. Processing Conditions

In general, higher cooking temperatures, longer cooking times, relative humidity, and heat transfer medium are the processing factors that impact the AA formation in foods [6, 90]. Several studies have discussed the impact of the time-temperature combination on AA formation. These researches illustrated those process parameters could be an effective way of AA minimization [91]. AA formation could be reduced by prolonged heating at low temperatures or by regulating the oven temperature as higher temperatures at the early stages followed by low temperatures at the final stage [92]. Prolonged heating at high temperatures generates low levels of AA due to degradation reactions [91, 92]. Thermal processes involve low temperatures or water (as boiling or steam cooking) as the medium known to reduce the Maillard reactions and accordingly decrease AA formation [91]. Apart from that, water activity also influences the rate of AA formation. It has been demonstrated that no AA was observed when water is evaporated from the food completely [90]. In contrast, high AA formation was observed at a given temperature with low moisture in the food [91]. Furthermore, high relative humidity during baking was proved to be effective for the reduction of AA in bakery products. Low temperatures are suitable for foods that have high residual moisture. Moreover, AA formation in cereal products that have low moisture could be reduced by using deck ovens that transfer heat by conduction and radiation, then convection ovens, which are based on forced air circulation. Thus, the forced air circulation is accountable for quicker drying, and subsequently, the surface was increased, which enhances AA formation [92].

### 5.2. Agronomic and Genetic Approaches

Agronomic and genetic factors in raw materials could be altered to get lower reducing sugars and asparagine content. According to the literature, sulfur deficiency leads to the greatest asparagine accumulation in cereal grain, and in conditions of severe sulfur deprivation, asparagine can accumulate up to 50% of the total free amino acid [93]. Additionally, nitrogen fertilization resulted in a high level of free asparagine [94]. Moreover, sprouting is also affecting AA formation in cereal grain, as it leads to the degradation of starch and proteins, and the release of sugars and asparagine [95]. Sprouting of potato tubers can be prevented by storage at 4°C, which results in cold sweetening in which starch is broken into reducing sugars. This can be overcome by storing at 12°C or spraying the harvest with chemical sprout suppressants [96]. Claus et al. [97] reported that bread prepared with sprouted grains had a 500% increment in AA compared to normal grains. Therefore, sprouted grains should not be used for bakery products [98, 99]. Furthermore, the maturity stage is also impacting the AA formation. Mature fruits comprise high reducing sugars than immature ones, which results in a higher AA formation [100].

Genetic modification is another way of minimizing AA in raw materials by the selection of cultivars and genetic factors such as raw materials with a low amount of asparagine and sugar levels. Asparaginase is the predominant enzyme involved in the hydrolysis of asparagine that results in forming of aspartate and ammonia [101]. Glutamine synthetase, asparagine synthetase, and asparaginase are the potential candidate genes that are involved in amino acid signaling either for manipulation by genetic modification or mutagenesis or for the development of genetic markers for breeding purposes [102].

### 5.3. Additives, Competitive Inhibitors, and Enzymes

Various types of additives, enzymes, competitive inhibitors, and plant extracts that have different inhibitory mechanisms can be used to mitigate AA formation [1, 103]. Sulfites have been confirmed to be effective, based on the inhibition of the intermediate compounds in AA formation [104]. Proteins or amino acids (soy protein hydrolysates and sulfur-containing amino acids) which competes with the precursors in food can be added to mitigate acrylamide formation; however, the mechanism depends on the nature of the amino acids [105]. The inhibitory effect of the sulfur-containing amino acids is based on a Michael-type addition (nucleophilic addition) reaction between the SH group of the sulfur amino acids and AA [106]. Cysteine and glutathione were effectively proven results for the reduction of AA in olive ripe, potatoes, and grape juice respectively [107, 108].

Asparaginase is the key enzyme that hydrolyzes asparagine into aspartic acid and ammonia thus removing the precursor of AA from the cereals, potatoes, and bakery products while keeping the sensory characteristics of the final product. Asparaginase does not exist in humans but naturally does in some bacteria (*E. coli*, Erwinia), mold, plants, and some animals. At present microbial asparaginases, those from *Aspergillus oryzae* and *Aspergillus niger* are commercially available [101]. The activity of the enzyme depends on the concentration of the enzyme, pH, incubation time, temperature, and product formulation. The action of asparaginase can be enhanced by combining it with another mitigation approach. Combine blanching with asparaginase immersion improves the AA reduction in potato chips [109].

Plant extracts consisting natural phenolics also show to have effective results for AA mitigation. Most of the plant extracts that include green tea, bamboo, mint, berry, grape extract, rosemary aqueous extract, oregano, and pomegranate flower have reported a significant decrease of AA [5, 110, 111]. Their inhibitory action mainly depends on phenolic compounds such as flavonoids, phenolic acids, and tannins [112], and their antioxidant power, carbonyl trapping activity, amino acid precipitation effect, and other supposed mechanisms of actions [113]. According to the literature, sometimes, antioxidants can either promote or reduce AA formation, irrespective of antioxidant power [5]. For example, naringenin, with weak antioxidant activity, can strongly block AA formation but curcumin as a potent antioxidant facilitates AA production [5, 114]. Moreover, sulfurous compounds like garlic and nitrogenous compound, piperine, weakly reduce AA in foods [115].

Organic acids such as citric, tartaric, acetic, and lactic acid were shown to have effective results in the mitigation of AA in gingerbread, biscuits, and potatoes by lowering the pH that creates unfavorable conditions [104]. Since acids lead to alterations of the organoleptic qualities and have synergistic effects, the added amount is limited. Several studies have indicated that B vitamins such as nicotinic acid (B3), pyridoxamine (B6), pyridoxine (B6), and biotin (B7) have shown some effective reduction of AA. Vitamin B3 was effective for fried potato strips; however, vitamin B7 and thiamine (B1) were not efficient in the reduction of AA in a mixture of potato flour and semolina [114, 115]. Recent studies found that hydrocolloids such as alginic acid and pectin decrease the amount of AA in fried potato strips [116]. In contrast, hydrocolloids such as carob gum, carrageenan, hydroxypropyl di starch phosphate, and xanthan gum increase the quantity of AA [117]. The inhibition mechanism is based on the molecular movements to make interactions between hydrocolloids and the precursors of AA [118].

### 5.4. Blanching

The blanching of potatoes before frying has been found very effective in minimizing AA [104]. Blanching helps to remove sugars from potatoes, thus reducing the number of AA precursors. Mestdagh et al. [119] reported that high-temperature short-time blanching proved to be more effective than blanching at low temperatures for a long time.

### 5.5. Fermentation

Fermentation with yeast has been reported to the consumption of the asparagine precursor content in bread [120]. Wang et al. [121] reported that about 40–60% asparagine can be reduced by yeast fermentation whereas the contents of reducing sugars are increased. Nevertheless, sourdough fermentation negatively affects the reduction of asparagine by yeast and leads to an increase in the AA [121]. Lactic acid fermentation in potatoes increases asparagine contents and reduces the contents of reducing sugars, which, accordingly, result in decreasing AA contents [122].

### 5.6. Encapsulation

Encapsulation is a new approach for AA mitigation, based on the controlled release of reactants during the processing time. The encapsulation of sodium chloride is an effective strategy to control the formation of some precursors of the Maillard browning [123]. Commonly, monovalent and divalent cations minimize the AA formation by sugar decomposition. Consequently, those cations alter the reaction pathway that leads to the formation of HMF or furan formation. Sodium ions determine the formation of fructofuranosyl cation which is one of the key precursors of HMF [124]. This strategy has been broadly illustrated in partially dehydrated cherry, tomato and grapefruits, cookies, and other bakery products [123–125]. Iron and ascorbic acids also can be used for encapsulation purposes [126]. AA formation is influenced by metal ions, through promoting oxidation reaction in the Maillard browning that leads to the formation of dicarbonyl compounds [127]. Nevertheless, in some cases, metal ions can suppress the Maillard reaction or can be effectively used to catalyze the precipitation and the successive removal of the brown materials, which leads to AA formation [126]. Ascorbic acid is more sensitive to oxidation and is implied in the formation of furan [127]. Encapsulation of these compounds in thermally processed food is thus interesting to preserve them from thermal degradation. The addition of flaxseed oil nanocapsules in bread protects them from oxidation which leads to an increase in the nutritional value of bread and less AA and HMF without affecting the sensory properties [128].

Postprocess approaches are now being used to eliminate produced AA during food processing, but it needs technological advancement to continue with high cost. Several studies (i) remove AA under stimulated gastrointestinal conditions using a dynamic system [129] and (ii) apply a hydration step followed by a vacuum drying for finished food with less moisture [19]. Most of the mitigation strategies directly affect the organoleptic properties; thus, a combination of two or more approaches may lead to improved mitigation efficiency and sensorial problems in food commodities.

## 6. Conclusion and Future Prospective

Acrylamide is a heat-induced toxic substance that was discovered in food commodities in 2002; factors that affect its formation, formation mechanisms, AA detection techniques, health impacts, and mitigation strategies were broadly discussed in this paper. For mitigation purposes, process parameters, novel, integrated processing techniques, use of additives, and different pre- and postprocessing treatments have been reported in several studies. Nevertheless, the same approach is not applicable in all food categories; it depends on food compositions and different product technologies. Also, there is no fully accepted extraction method of AA from foodstuffs. Further, more studies will have to be conducted to investigate the extraction methods of different kinds of foods on AA.

The Food and Agriculture Organization (FAO) and WHO have found a global network to permit all interested parties to communicate information with regard to ongoing studies on acrylamide in food. According to the European Food Safety Agency, areas of tumors, dose-response, and mode of action for each of the tumors, identified in animal test species, species sensitivity, physiologically based pharmacokinetic (PBPK) modeling, epidemiological studies on additional populations or specific groups of the population, and additional cancer sites to draw firm conclusions on the cancer risk associated to acrylamide exposure should be investigated. Due to the very limited data on dietary exposure, actual acrylamide exposures should be validated by the Food Frequency Questionnaires (FFQ) coupled with biomarkers.

HMF is also a heat-induced toxic intermediate that participates in the formation of AA in the food matrix. Investigation of the extent of HMF and related carcinogenicity in animals, epidemiological data associated with dietary exposure to HMF, and increased cancer risk mitigation approaches to reduce HMF content in foods should be further studied.

Moreover, community awareness about the dangers of acrylamide along with possible ways to reduce its formation needs to be increased. Furthermore, government agencies and the industry about the dangers and possible ways of reducing this food contaminant must be provided. Most of the mitigation strategies were investigated to reduce acrylamide under the research level. Thus, implementing suitable mitigation strategies at the industrial scale and applying good practices for household cooking or changing food behaviors and consumption patterns are most important to eliminate the adverse consequence of acrylamide.

## Figures and Tables

**Table 1:** Summary of reported AA extraction and derivatization methods in different food products.

Food sample	Extraction and analysis method	Reference
French fries	Extraction with water and subjected to solid-phase extraction (SPE). Derivatization with brominating reagent (KBr, HBr, and Br2 water)	[55]
	Extraction with water and 1,2-dichloroethane Derivatization with xanthydrol solution and 1 mL of HCl at 40°C in the dark, then slightly alkalinized with KOH and followed by saturated NaCl. Then, AA derivative was extracted twice with EtAc.	[56]

Bakery products	Extracted with water and derivatized with the brominating reagent	[57]
	Extracted with water-methanol (80 : 20) Derivatization with xanthydrol and 1 mL HCl at 40°C in the dark AA derivative was extracted with ethylene tetrachloride and methanol. Then directly injected into the GC-MS.	[58]

Cereals	The sample was mixed with water and shaken to get a soft dough. IS and KOH: ethanol (80 : 20) was added and centrifuged followed by the upper clear aqueous phase was separated. Derivatization with xanthydrol and 1 mL HCl at 40°C in the dark. AA derivative was extracted using Dispersive liquid-liquid microextraction with tetrachloroethylene as extracting solvent and ethanol as a dispersing solvent, then directly injected into the GC-MS.	[59]
	The sample was spiked with IS and added distilled water, homogenized, and acidified to pH 4–5 by glacial acetic acid, and Carrez reagent was added, then centrifuged, and the supernatant was filtered, derivatized with calcinated KBr, HBr, and saturated Br_2_.	[60]

Coffee	Extraction was done by using MSPD. Derivatization with the brominating reagent	[61]

Baby foods	The sample was defatted with n-hexane and vortexed. The supernatant was removed by filtration. Then, the solid was dried and spiked with IS, and the aqueous layer was filtered through a syringe filter. Derivatized with the brominating reagent	[62]

Tomato	Extraction with water and derivatize with the brominating reagent	[63, 64]

Green tea	Extraction with water and subjected to SPE. Derivatization with the brominating reagent	[65]

Commercial frying oil	Extraction with water and defatting with hexane, followed by being subjected to SPE. Derivatization with xanthydrol solution and 1 mL of HCl at 40°C in the dark, then slightly alkalinized with KOH and followed by saturated NaCl. Then, AA derivative was extracted twice with EtAc.	[66]

**Table 2:** AA content in different food varieties.

Food sample	AA level	Reference
Potato crisps	325 *μ*g/kg	[67]
Breakfast cereals	<62–803 *μ*g/kg	[68]
Carbohydrate-rich food	< 20–2,528 *μ*g/kg	[69]
Pizza, minced meat, and fried bacon	0–1,480 *μ*g/kg	[70]
Espresso coffee	11.4–36.2 *μ*g/L	[71]
Chocolate	102 *μ*g/kg	[72]
Green tea	30-56 *μ*g/kg	[65].
Baby foods	2-516 *μ*g/kg	[73]
Tomato	50 *μ*g/kg-124 *μ*g/kg	[73]

## Data Availability

The numerical data used to support the findings of this study are available from the corresponding author upon request.
